# A brave new MYC-amplified world

**DOI:** 10.18632/aging.100777

**Published:** 2015-07-08

**Authors:** Marc S. Weinberg, Jonathan R. Hart, Peter K. Vogt

**Affiliations:** Department of Molecular and Experimental Medicine, The Scripps Research Institute, La Jolla, CA, USA

MYC is a basic domain helix-loop-helix leucine zipper (bHLH-LZ) transcriptional regulator that forms a heterodimer with the related but much smaller bHLH-LZ protein Max to bind sequence-specifically to DNA. In the last few years, the understanding of MYC action has undergone a complete transformation. Originally thought of as a conventional transcription factor with a large but limited number of distinct target genes, MYC has emerged as a transcriptional amplifier, whereby global transcription is tuned to specific cellular levels of MYC. Evidence for this universal involvement of MYC in transcription was originally obtained in studies of the protein-coding transcriptome [1, 2]. However, recent papers now extend MYC's broad activity to the entire non-coding transcriptome, showing that all non-coding RNAs are subject to MYC regulation [3, 4]. These data are in accord with the role of MYC as general transcriptional amplifier.

It has been known for some time that MYC can affect the transcription of short noncoding RNAs such as microRNAs [5]. However, these constitute only a small minority of all noncoding RNAs. A larger and far less explored segment of the non-coding transcriptome is made up of long non-coding RNAs (lncRNAs) which typically form functional secondary and higher order structures comprising protein-protein or protein-nucleic acid complexes [6, 7]. Functions and regulation of lncRNAs largely are virgin, unexplored territory.

The reach of MYC to all of the non-coding transcriptome constitutes a huge, new expansion of MYC's sphere of influence. Considering that the vast majority of all transcribed sequences is non-coding, their inclusion among the transcriptional clients of MYC amounts to multiplying the MYC universe.

The effect of MYC on non-coding transcripts is correlated with MYC binding to promoter-proximal sites and therefore appears to be a direct function of MYC, not mediated by a different downstream transcriptional regulator. Although MYC binding to transcriptional start sites is identical in different cell types, the resulting expression patterns are cell type-specific. This surprising observation is unexplained at this time and requires further analysis. As is the case with the coding transcriptome, MYC can function as a positive as well as negative regulator of transcription. The genome-wide effects of MYC on the non-coding transcriptome were discovered by RNAseq analysis. However, for selected genes, these data have been independently validated for specific lncRNAs by determining steady-state RNA levels by qRT-PCR and direct transcriptional effects by nuclear run-on experiments. This data is augmented by visualization with global epigenetic and MYC ChIP data.

**Figure 1:**
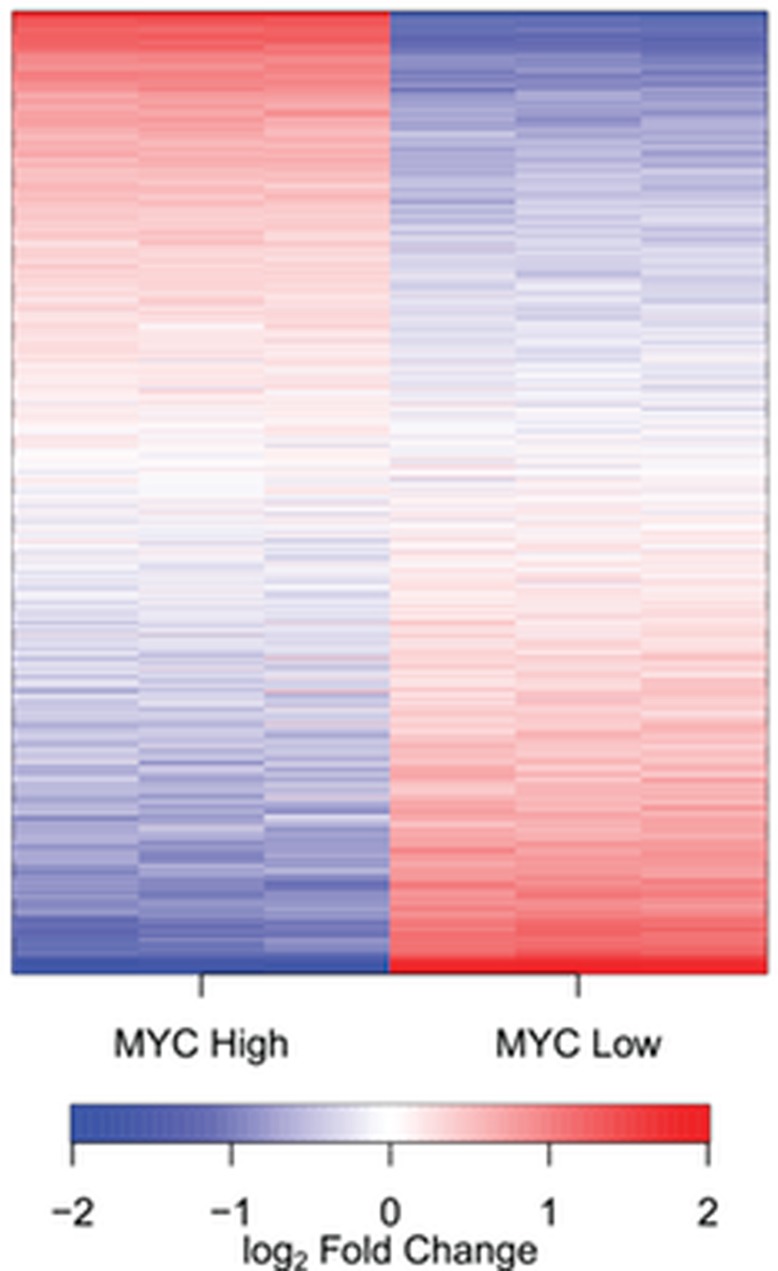
MYC regulates the expression of noncoding (nc) RNAs A heatmap of ncRNAs detected in the human B cell line P493–6. P493–6 cells were analyzed by RNAseq in triplicate under conditions of high and low MYC expression. Transcripts are ordered by the change in expression upon MYC upregulation, with those most upregulated at the top and those most downregulated at the bottom. The figure shows that the expression of virtually all ncRNAs is affected by MYC; they are either upregulated or downregulated. There extremely few if any noncoding transcripts that do not respond to MYC.

The emergent importance of the non-coding transcriptome leads to the necessary question of how this class of RNA is transcriptionally regulated. MYC can now be considered to be an important, but not the only player, in this network of key regulators. We are at the cusp of a much more detailed analysis of regulatory mechanisms. MYC is also arguably the "Emperor of Oncogenes", involved as a driving force in most and probably all human cancers. A complete understanding of the mechanisms by which MYC achieves these oncogenic effects remains a daunting challenge, a challenge that has been considerably magnified by the regulatory role of MYC in the noncoding transcriptome. Exciting new genome editing and modulatory technologies will likely be adopted to generate a first compendium of cancer-relevant lncRNAs, while providing hints of their underlying function in the cell. It is a task that promises to provide critical new insights into the molecular biology of cancer.

## References

[1] Lin CY (2012). Cell.

[2] Nie Z (2012). Cell.

[3] Hart JR (2014). Oncotarget.

[4] Kim T (2015). Journal of the National Cancer Institute.

[5] Cairo S (2010). Proceedings of the National Academy of Sciences of the United States of America.

[6] Roberts TC (2014). Epigenetics: official journal of the DNA Methylation Society.

[7] Morris KV, Mattick JS (2014). Nature reviews Genetics.

